# Shelf‐stable, ready‐to‐use therapeutic patches: Dip‐and‐deliver solutions for personalized wound care

**DOI:** 10.1002/btm2.70131

**Published:** 2026-03-23

**Authors:** Ameya P. Chaudhari, Parmiss Khosravi, Zajeba Tabashsum, Hattie E. Hensley, Claire J. Wang, Emilie A. Moses, Samantha Harris, Anthony Hazelton, Palas B. Tiwade, Rachel VanKeulen‐Miller, Owen S. Fenton, Sergei S. Sheiko, Sarah E. Rowe, Juliane Nguyen

**Affiliations:** ^1^ Division of Pharmacoengineering and Molecular Pharmaceutics Eshelman School of Pharmacy, University of North Carolina at Chapel Hill Chapel Hill North Carolina USA; ^2^ Lampe Joint Department of Biomedical Engineering University of North Carolina at Chapel Hill Chapel Hill North Carolina USA; ^3^ Department of Microbiology and Immunology University of North Carolina‐Chapel Hill Chapel Hill North Carolina USA; ^4^ Department of Chemistry University of North Carolina at Chapel Hill Chapel Hill North Carolina USA; ^5^ Department of Pharmacology School of Medicine, University of North Carolina at Chapel Hill Chapel Hill North Carolina USA; ^6^ UNC McAllister Heart Institute, University of North Carolina at Chapel Hill Chapel Hill North Carolina USA

## Abstract

Chronic wounds often require advanced dressing materials that can conform to complex skin movements and maintain therapeutic efficacy. Conventional dressings are typically non‐adhesive and non‐auxetic, necessitating external fixation and limiting their mechanical compatibility with natural tissue deformation. To overcome these challenges, we developed an auxetic skin mesh platform with instant adhesion, enabling improved mechanical conformity and ability to mimic skin movement. In this study, we developed a shelf‐stable auxetic skin mesh capable of loading and releasing a broad range of therapeutics through a simple dip‐and‐deliver process. We compared air‐dried and freeze‐dried meshes against unprocessed controls to evaluate mechanical strength, adhesion, porosity, and drug‐release performance. The platform effectively delivered diverse cargos, including lipid nanoparticles (LNPs), extracellular vesicles (EVs), proteins, small molecules, and FDA‐approved drugs such as platelet‐derived growth factor (PDGF‐BB) and antibiotics, demonstrating controlled release and enhanced angiogenesis in a diabetic mouse model. These results highlight the mesh's versatility as a bioadhesive drug‐delivery system with strong potential to improve wound healing outcomes.

**Translational Impact Statement**

Current standard‐of‐care dressings for diabetic foot ulcers are non‐adhesive and non‐auxetic, often requiring staples or sutures for fixation and are prone to premature detachment, leading to poor patient compliance and delayed healing. We introduce shelf‐stable, skin‐mimetic meshes that are instantly adhesive and auxetic, enabling ability to conform to natural skin movements. These meshes can be rehydrated with a therapeutic agent of choice and deliver drugs in a controlled‐release manner. By combining mechanical adaptability and controlled drug delivery, this platform offers a clinically translatable solution to enhance patient compliance and diabetic wound healing.

## 1 INTRODUCTION

Diabetic foot ulcers (DFUs) affect 20 million individuals worldwide each year,^1^ with a significant percentage of patients ultimately requiring lower limb amputation.^2^, ^3^ This condition is not only physically debilitating but also severely impacts patients' quality of life. The current standard of care consists of performing tissue debridement, followed by the application of extracellular matrix (ECM)‐based dressings.^4^, ^5^ Nonetheless, fewer than two‐thirds of the patient population respond to these treatments.^6^ Products such as the Integra® Bilayer Wound Matrix and the Oasis® Ultra Tri‐Layer Wound Matrix have been employed to promote and accelerate wound healing in DFUs. However, these dressings are not inherently adhesive and require stapling or suturing to the wound bed, causing discomfort and pain during dressing changes, especially in the elderly patient population.^7^, ^8^

**FIGURE 1:**
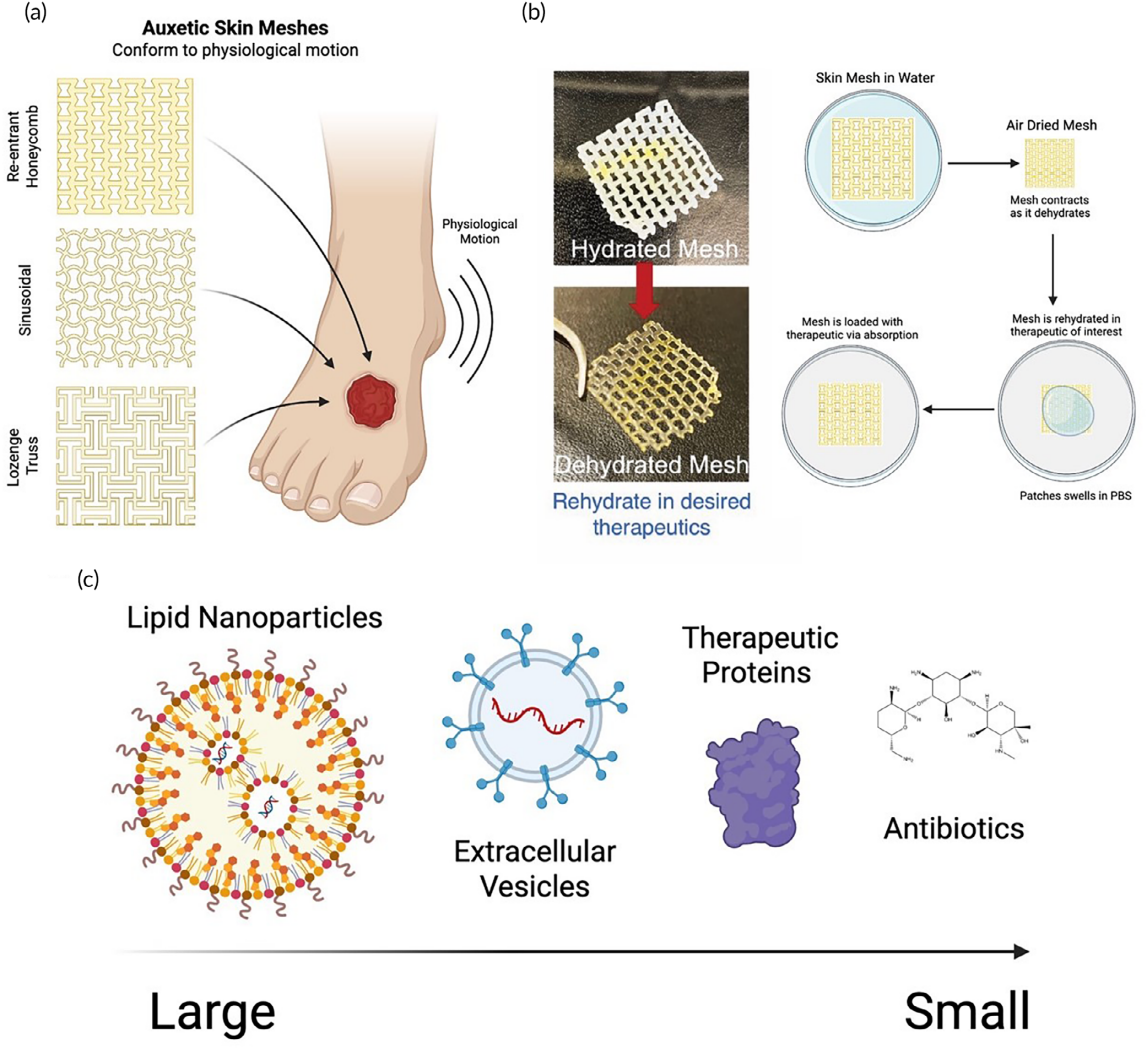
Dip‐and‐deliver, shelf‐stable, auxetic skin meshes for loading various therapeutics. (a) Auxetic skin meshes conform to skin movement reducing premature breakage and detachment. (b) Air‐dried meshes for extended shelf life and room temperature storage and schematic illustrating the dip‐and‐deliver workflow: Dehydration of the skin mesh followed by rehydration in a therapeutic solution of choice. (c) The range of therapeutics that can be loaded onto the skin meshes.

To address these limitations, there is a need for a wound dressing that is both self‐adhering and biodegradable. In our previous work, we developed an auxetic skin mesh platform that meets these criteria. This bioadhesive and biodegradable mesh is designed to conform to the dynamic mechanical environment of human skin, which undergoes continuous stretching, flexing, and movement.^9^, ^10^, ^11^, ^12^ Traditional, non‐auxetic structures are prone to fatigue under such repetitive motion, leading to premature detachment or failure. In contrast, auxetic structures, characterized by a negative Poisson's ratio, expand and contract uniformly in all directions, allowing the mesh to better conform to the skin's natural movements (Figure 1a).^13^, ^14^ The meshes were previously optimized and synthesized using gelatin methacrylate (GelMA) and acrylic acid. GelMA was selected for its biocompatibility, biodegradability, and tunable mechanical properties, which enable adjustment of hydrogel stiffness as required.^15^, ^16^ Additionally, gelatin contains RGD peptide sequences, derived from collagen, that promote cellular adhesion and proliferation, making it an excellent candidate for wound healing applications.^17^ Furthermore, the acrylic acid content was optimized to achieve suitable stiffness for biological use and to enhance mesh bioadhesion through hydrogen bonding.^9^

In the present study, we aimed to enhance the translational potential of our technology. Specifically, we investigated dehydration methods to render the patches (also known as skin meshes) shelf‐stable, enabling storage at room temperature without the need for cold chain logistics (Figure 1b). Additionally, we explored the capacity of our skin meshes to load and release therapeutic agents in a sustained manner. We characterized the release kinetics of both small and large molecules, including drug carriers such as lipid nanoparticles (LNPs) and extracellular vesicles (EVs) (Figure 1c). We demonstrated that drugs loaded onto our meshes remained functionally active, promoting fibroblast proliferation and inhibiting the growth of DFU‐relevant bacteria in vitro. Finally, we evaluated the efficacy of platelet‐derived growth factor (PDGF)‐loaded skin meshes in a diabetic wound healing mouse model. Our findings show that these skin meshes not only promotes angiogenesis but also offer practical advantages in terms of storage and point‐of‐care drug loading.

In summary, we have developed a versatile and clinically relevant auxetic wound dressing platform that is shelf‐stable, bioadhesive, biodegradable, and capable of delivering therapeutics tailored to individual patient needs.

## 2 METHODS

### 2.1 Regulatory

All the animal studies were approved by the Institutional Animal Care and Use Committee (approval ID: 24‐029.0) at the University of North Carolina at Chapel Hill.

### 2.2 Materials

GelMA 40% (Cat. No. 900629), LAP (Cat. No. 900889) and acrylic acid (Cat. No. 147230) were purchased from Sigma Aldrich. Mouse PDGF‐BB Recombinant Protein (Cat. No. 315‐18‐250UG) was purchased from Thermofisher. Ovalbumin, Fluorescein Conjugate (Cat. No. O23020) was purchased from Invitrogen. Cholesterol, phosphate‐buffered saline (PBS, pH 7.4), sodium citrate, Dulbecco's phosphate‐buffered saline (DPBS), ethanol, and Triton X‐100 were obtained from Sigma‐Aldrich. The lipids 1,2‐distearoyl‐sn‐glycero‐3‐phosphocholine (DSPC) and 1,2‐dimyristoyl‐rac‐glycero‐3‐methoxypolyethylene glycol‐2000 (DMG PEG 2000) were sourced from Avanti Polar Lipids. SM‐102 was purchased from Broadpharm. The Quant‐iT RiboGreen RNA assay kit and Slide‐A‐Lyzer™ Dialysis Cassettes (20 K MWCO, 0.5 mL) were acquired from Thermo Fisher Scientific (USA).

### 2.3 Skin mesh printing and postprocessing

Skin meshes were printed as described previously.^9^ Briefly, 100 μL of acrylic acid was added to 400 μL of 10% w/v GelMA solution. LAP was added to achieve a final concentration of 0.03% w/v LAP photo initiator and 2 μL of yellow food dye. The printing ink was mixed thoroughly and poured into a 30 × 30 mm reservoir made on a PDMS‐coated petri dish. The petri dish was then mounted on the digital light projection system (LumenX, Cellink AB), and the ink was exposed to the UV light for 20 mW/cm^2^ for 2 min. For post‐processing, the skin meshes were incubated in 0.5 M calcium chloride and 0.05 M sodium hydroxide solution. The skin meshes were then incubated in water overnight at 4°C to wash away LAP and the yellow food dye. For air‐dried meshes, the skin meshes were allowed to air dry overnight within a biosafety cabinet at room temperature. For freeze‐dried meshes, they were flash frozen in liquid nitrogen and then freeze‐dried in a lyophilizer.

### 2.4 Tensile tests

As‐synthesized wet unprocessed hydrogel films, rehydrated air‐dried, and rehydrated freeze‐dried films were incubated in 0.5 mM CaCl_2_ for 5 min and subjected to uniaxial extension experiments on an RSA‐G2 dynamic mechanical analyzer (DMA) from TA Instruments with a constant strain rate of 0.005 s^−1^. Dog‐bone shaped samples with a width of 2 mm, length of 12 mm, and thickness of 1 mm were extended until break. All samples were run in triplicate to verify reproducibility.

### 2.5 Force of detachment tests

**FIGURE 2:**
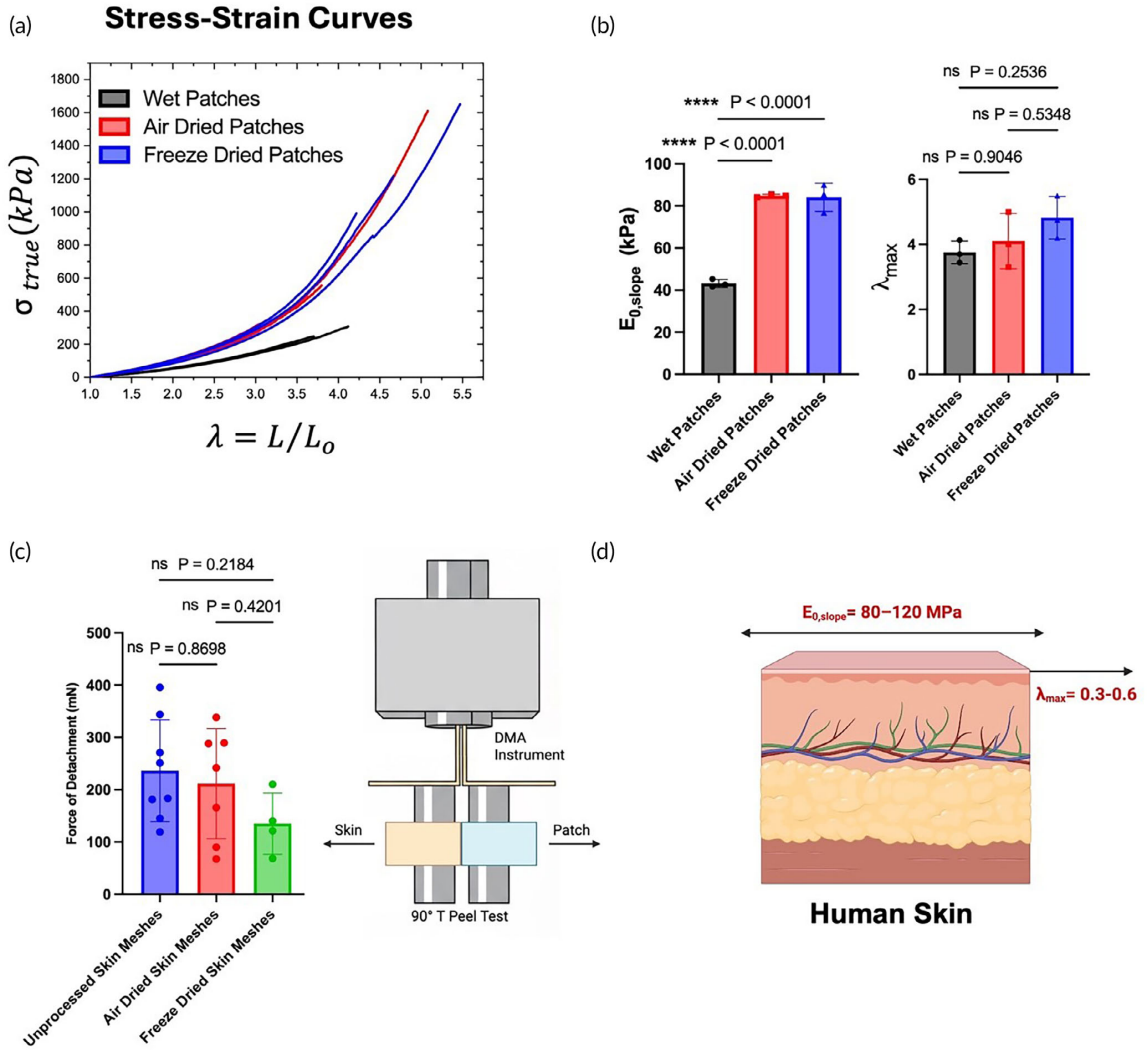
Effects of dehydration on mechanical properties and adhesion. (a) Stress–Strain curves of wet (unprocessed), air‐dried, and freeze‐dried mesh show that the drying process affects the tensile response. (b) Young's modulus (*E*_0_) and elongation‐at‐break (λ_max_) of wet meshes compared to air‐dried and freeze‐dried meshes. One‐way ANOVA was performed, followed by Tukey's post and Šidák hoc test for multiple comparisons respectively (*n* = 3 per experimental group). (c) Detachment force required to remove the meshes from the tissue. One‐way ANOVA was performed, followed by Tukey's post hoc test for multiple comparisons (Unprocessed *n* = 8, Air‐Dried *n* = 7, Freeze‐Dried *n* = 4). (d) Schematic depicting the elastic modulus and yield strain of human skin. Data is represented as mean ± SD.

Adhesion experiments were performed on wet (unprocessed), air‐dried, and freeze‐dried hydrogel films on chicken muscle tissue using a TA Instruments HR30 Hybrid DMA/Rheometer with a TA Instruments extensional viscosity (EVA) geometry at a constant strain rate of 0.05 mm/s^−1^. The EVA fixture is also known as the Sentmanat Extensional Rheometer (SER) fixture. Chicken muscle tissue was secured in one drum of the EVA and the film 10 mm in width and 20 mm in length was adhered to the tissue. The remaining unadhered film was secured to the other drum (Figure 2c). 90°T‐peel experiments were performed to determine the force of detachment required to detach the film from the tissue.

### 2.6 SEM method

An auxetic skin mesh was air‐dried for 24 h in preparation for scanning electron microscopy (SEM) analysis. To image the auxetic skin mesh in its hydrated state, the sample was prepared using critical point drying. The surface and cross‐section morphology of the mesh were studied using the Hitachi S‐4700 Cold Cathode Field Emission SEM at various magnifications. The microstructure and surface porosity of the auxetic skin mesh were measured using ImageJ software.

### 2.7 Fourier‐transform infrared spectroscopy (FTIR)

FTIR data was taken on a Bruker Hyperion microscope attached to a Tensor 27 spectrometer. Data were acquired with a single bounce Ge ATR objective that mounts onto the Hyperion microscope. The ATR crystal contacted the sample with a spot that was ~100 μm in diameter. The spectral resolution was 4 cm^−1^, and 32 scans were averaged for the sample and the blank. The water and CO_2_ compensation feature in the Bruker OPUS software was used to minimize the effect of these peaks on the spectra, and baselines were manually subtracted. Spectra were normalized to the peak ~1700 cm^−1^ by dividing each spectrum by the intensity at that datapoint.

### 2.8 Extracellular vesicle (EV) isolation and characterization

Conditioned media (CM) from HEK293 cells was collected, and EVs were isolated via differential ultracentrifugation, as previously described.^18^, ^19^, ^20^, ^21^ Briefly, CM was sequentially centrifuged at 500 × g for 10 min, 2000 × g for 10 min, and 10,000 × g for 30 min to remove cells and debris. The supernatant was then filtered through a 0.22 μm membrane and subjected to ultracentrifugation at 100,000 × g for 80 min at 4°C. The resulting EV pellet was resuspended in 0.02 μm‐filtered phosphate‐buffered saline (PBS). EVs were labeled with the lipophilic dye DiD at a final concentration of 1.5 μM, followed by washing with PBS and ultracentrifugation. EV size distribution and particle concentration were determined using nanoparticle tracking analysis (NTA) on a ZetaView instrument (Particle Metrix).

### 2.9 Lipid nanoparticle (LNP) synthesis and characterization

Lipid nanoparticles (LNPs) were synthesized following the Moderna formulation. Briefly, the ionizable lipid SM‐102, DSPC, cholesterol, DMG‐PEG2000, and the lipophilic dye DiD were dissolved in ethanol at a molar ratio of 49:10:38.5:1.5:1, respectively. Firefly luciferase mRNA was dissolved in 10 mM citrate buffer (pH 4.0). The lipid and RNA solutions were combined at a lipid‐to‐RNA weight ratio of 10:1 using a microfluidic mixing device, with flow rates of 900 μL/min for the lipid phase and 300 μL/min for the aqueous phase. Following synthesis, the LNP suspension was dialyzed against phosphate‐buffered saline (PBS) using a 20 kDa molecular weight cutoff Slide‐A‐Lyzer at 4°C for 2 h to remove ethanol and unencapsulated components. Particle concentrations were assessed using nanoparticle tracking analysis (NTA). LNPs were diluted 1:200 in 1× phosphate‐buffered saline (PBS) for size and polydispersity index (PDI) measurements using the NanoBrook 90 Plus Zeta instrument (Brookhaven Instruments, USA). For surface charge analysis, LNPs were similarly diluted 1:200 in 0.1× PBS and assessed with the same instrument. To determine encapsulation efficiency and total mRNA content, the Quant‐iT RiboGreen Assay Kit was employed following the manufacturer's protocol, both in the presence and absence of lysis using a 2% Triton X‐100 solution.

### 2.10 Drug loading and release studies

Hydrogel discs were prepared using an 8 mm biopsy punch and subsequently divided into two groups: wet (unprocessed) and air‐dried for 24 h. Drug loading was performed by directly applying the drug solution onto the surface of the meshes. Release profiles were evaluated using fluorophore‐conjugated drugs or fluorophore‐labeled drug carriers. The protein release kinetics were assessed using fluorescein isothiocyanate (FITC)‐labeled ovalbumin (OVA‐FITC) at three different dosages: 2 μg, 5 μg, and 10 μg. For antibiotic release studies, Texas Red‐conjugated gentamicin was employed to monitor both loading efficiency and release behavior. The conjugate was synthesized by reacting Texas Red‐succinimidyl ester (TR‐SE) (Invitrogen) with gentamicin. TR‐SE was dissolved in N, N‐dimethylformamide at a concentration of 20 mg/mL. Gentamicin was dissolved in 100 mM potassium carbonate (pH 8.5) at 10 mg/mL concentration. TR‐SE was added to the gentamicin to dilute it to a final concentration of 0.57 mg/mL. They were reacted at 4°C for 30 min prior to use. Additionally, EVs and LNPs labeled with the lipophilic dye DiD were used to evaluate their respective loading and release profiles.

To evaluate release kinetics, drug‐ or carrier‐loaded discs were incubated in phosphate‐buffered saline (PBS) at 37 °C. The release medium was collected and replaced with fresh PBS at predetermined time points. The amount of released drug or carrier was quantified using a SpectraMax iD3 microplate reader, based on the fluorescence signal of the respective fluorophore‐labeled compounds. To assess loading capacity and efficiency, discs were washed with PBS after drug or carrier application. The amount of unbound drug or carrier in the wash solution was quantified using fluorescence measurements. PGDF‐BB release was assessed using a mouse PDGF‐BB ELISA Kit (Invitrogen). Loading efficiency was calculated by comparing the initial amount applied to the amount recovered in the wash, while loading capacity was determined based on the amount retained within the disc.

### 2.11 Zone of inhibition‐ in vitro efficacy of skin meshes

Overnight cultures of methicillin‐resistant *Staphylococcus aureus* (MRSA) strain JE2 and *Pseudomonas aeruginosa* PAO1 were grown in tryptic soy broth (TSB) or Luria‐Bertani (LB) broth, respectively at 37°C with shaking. On day 1, 100 μL of each overnight culture was evenly spread onto tryptic soy agar (TSA) for MRSA or LB agar plates for *P. aeruginosa*, using sterile cotton swabs (Q‐tips) to create a uniform bacterial lawn. Sterile absorbent hydrogel discs (dry or hydrated), sterile paper discs were placed at the freshly inoculated agar plates, and 10 μL of gentamicin (1 mg/mL) was pipetted directly onto each disc. For the drop application, 10 μL of gentamicin was added directly to the agar surface of the plate, without a paper disc or hydrogel. For swab application, sterile Q‐tips were dipped into the gentamicin solution and then used to swab the top of agar surface. As for the control, sterile absorbent hydrogel discs (dry or hydrated) were used. All plates were incubated at 37°C for 18 h. After incubation, paper discs or hydrogel discs were carefully removed, and zones of clearance (zones of inhibition) were measured to assess antimicrobial activity. On days 2 and 3, paper discs or hydrogel discs from the previous day's plates were reused. These were transferred to freshly inoculated agar plates and rehydrated with 100 μL of phosphate‐buffered saline (PBS) prior to incubation. Plates were then incubated under the same conditions, and zones of clearance were measured following incubation.

### 2.12 NIH 3T3 proliferation assay

A NIH 3T3 fibroblast proliferation assay was performed to assess the functional stability of PDGF‐BB following its loading onto the mesh. PDGF‐BB was loaded onto the skin mesh as previously described. The loaded meshes were then incubated in serum‐free Dulbecco's Modified Eagle Medium (DMEM) for 24 h at 37 °C to allow for protein release. After incubation, the conditioned medium was centrifuged at 2000 × g for 10 min to remove any residual debris from the skin mesh. The supernatant was then applied to NIH 3T3 cells cultured in 96‐well plates. After 24 h of incubation, the medium was aspirated, and the plates were frozen at −80 °C. To quantify cellular proliferation, the CyQUANT Cell Proliferation Assay Kit was used. Briefly, cells were lysed, and fluorescence intensity was measured to quantify the number of cells per well.

### 2.13 In vivo diabetic wound healing model

Six‐weeks old, female SKH‐1 mice were purchased from Charles River Laboratory. To induce diabetes, 65 mg/kg of Streptozotocin (STZ) along with 200 μL of 25% glucose was administered intraperitoneally daily for 5 days. Weights and glucose levels were monitored for a week. Mice with 250 mg/dL of glucose were considered diabetic. General anesthesia was induced using 4% isoflurane delivered in a gas mixture of 95% oxygen and 5% carbon dioxide at a flow rate of 1 L per minute. Anesthesia was maintained with 1–3% isoflurane. A 4 mm full‐thickness circular wound was surgically created on one side of the left flank. For analgesia, meloxicam (Pivetal) was administered subcutaneously at a dose of 5 mg/kg once daily for three consecutive days. The treatments were added, and Tegaderm was attached over the treatment to avoid cage mates taking the skin mesh off. The PDGF‐BB group received 10 μL of PDGF‐BB solution topically. The PDGF‐BB loaded meshes were prepared by incubating the meshes with PDGF‐BB solution (0.2 mg/mL) and allowing the mesh to be loaded with PDGF‐BB. New treatments were applied every 2 days, and wound tissue was harvested on day 9 to assess angiogenesis.

### 2.14 CD31 immunofluorescence staining

Formalin‐fixed, paraffin‐embedded (FFPE) skin sections were mounted onto positively charged slides and subjected to immunofluorescence (IF) staining using the CD31 antibody (Cell Signaling Technologies, 77699S). All staining procedures were performed on the Leica Bond III fully automated slide staining system (Leica Biosystems) utilizing the Bond Research Detection Kit (DS9455). Slides were initially hydrated in Bond Wash solution (AR9590), followed by antigen retrieval using Bond Epitope Retrieval Solution 1, pH 6.0 (AR9961). After pretreatment, tissue sections were blocked and incubated for 1 h with the CD31 primary antibody diluted at 1:200. Detection was achieved using the ready‐to‐use Novolink Polymer secondary antibody (Leica Biosystems, RE7260‐CE), followed by visualization with TSA Cy5 (Akoya Biosciences, SAT705A001EA). Nuclear counterstaining was performed using Hoechst 33258 (Invitrogen), and slides were cover‐slipped with ProLong Gold antifade reagent (Thermo Fisher Scientific, P36930). Staining run included a positive control.

Digital slide scanning was conducted using the Aperio ScanScope FL (Aperio Technologies Inc). Images were acquired in each fluorescence channel using a 20× objective lens (0.468 μm/pixel resolution) with line‐scan camera technology (U.S. Patent 6,711,283). Quantification of CD31‐positive vessels was performed using a pixel classifier trained with an Artificial Neural Network‐Multilayer Perceptron (ANN‐MLP) model implemented in QuPath. Briefly, the pixel classifier was trained on training images comprised of multiple subsections of skin tissue that were manually annotated to detect features like CD31+ cells (blood vessels) and background.^22^ The annotations produced by the pixel classifier were verified by visual inspection to ensure accurate detection. This pixel classifier was then applied to wound bed sections, where the classifier detected CD31+ blood vessels. Based on these detections, the number of blood vessel per unit area of tissue section and area of blood vessels per unit area of tissue section was calculated.

### 2.15 AI Statement

Microsoft Copilot was used to improve flow and readability of the sections. All AI‐assisted content was verified and edited by the authors to ensure accuracy and integrity.

## 3 RESULTS

### 3.1 Mechanical characterization of dried auxetic skin meshes

To enhance the clinical translatability of our meshes, we aimed to eliminate the need for cold‐chain storage by developing a shelf‐stable product that can be stored at room temperature. To achieve this, we implemented dehydration strategies to prevent mesh degradation over time. Specifically, two approaches were evaluated: air drying and freeze drying. Our objective was to compare the effects of these drying methods on the mechanical properties and determine which technique is most suitable for wound healing applications.

To investigate the impact of drying and rehydration on the mechanical properties of skin meshes, we conducted tensile tests (Figure 2a). The meshes were either air‐dried or freeze‐dried, then rehydrated in water and incubated in 0.5 M CaCl₂ for 5 min prior to mechanical testing.

As shown in Figure 2b, the drying process led to an increase in the elastic modulus of the meshes. This enhancement is likely due to the formation of hydrogen bonds within the mesh matrix upon dehydration, which may contribute to improved fracture and fatigue resistance.

Notably, native skin exhibits an elastic modulus ranging from 80 ‐ 120 MPa longitudinally and 40–70 MPa transversely, several orders of magnitude higher than that of the skin mesh.^12^ This ensures that the mesh will not impede natural skin movement and can conform seamlessly to dynamic skin deformation.

All the meshes, unprocessed and rehydrated, demonstrated high extensibility, with maximum yield strains exceeding 350%. Although dehydration appeared to increase mesh stiffness, no statistically significant change was observed in maximum yield strain. In contrast, native skin typically deforms up to 20–40% longitudinally and 30–60% transversely, indicating that the meshes can accommodate skin movement without mechanical mismatch, making them suitable for skin applications (Figure 2d).^12^

Finally, we assessed the effect of dehydration on the adhesive properties of the meshes. Meshes were applied to tissue samples, and the detachment force was measured. The unprocessed and air‐dried meshes required higher detachment forces compared to freeze‐dried meshes; however, no statistical significance was observed (Figure 2c). The reduced adhesion observed in freeze‐dried meshes may be attributed to microfractures and structural damage caused by ice crystal formation during the freezing process.

### 3.2 Structural characterization of the skin meshes

**FIGURE 3:**
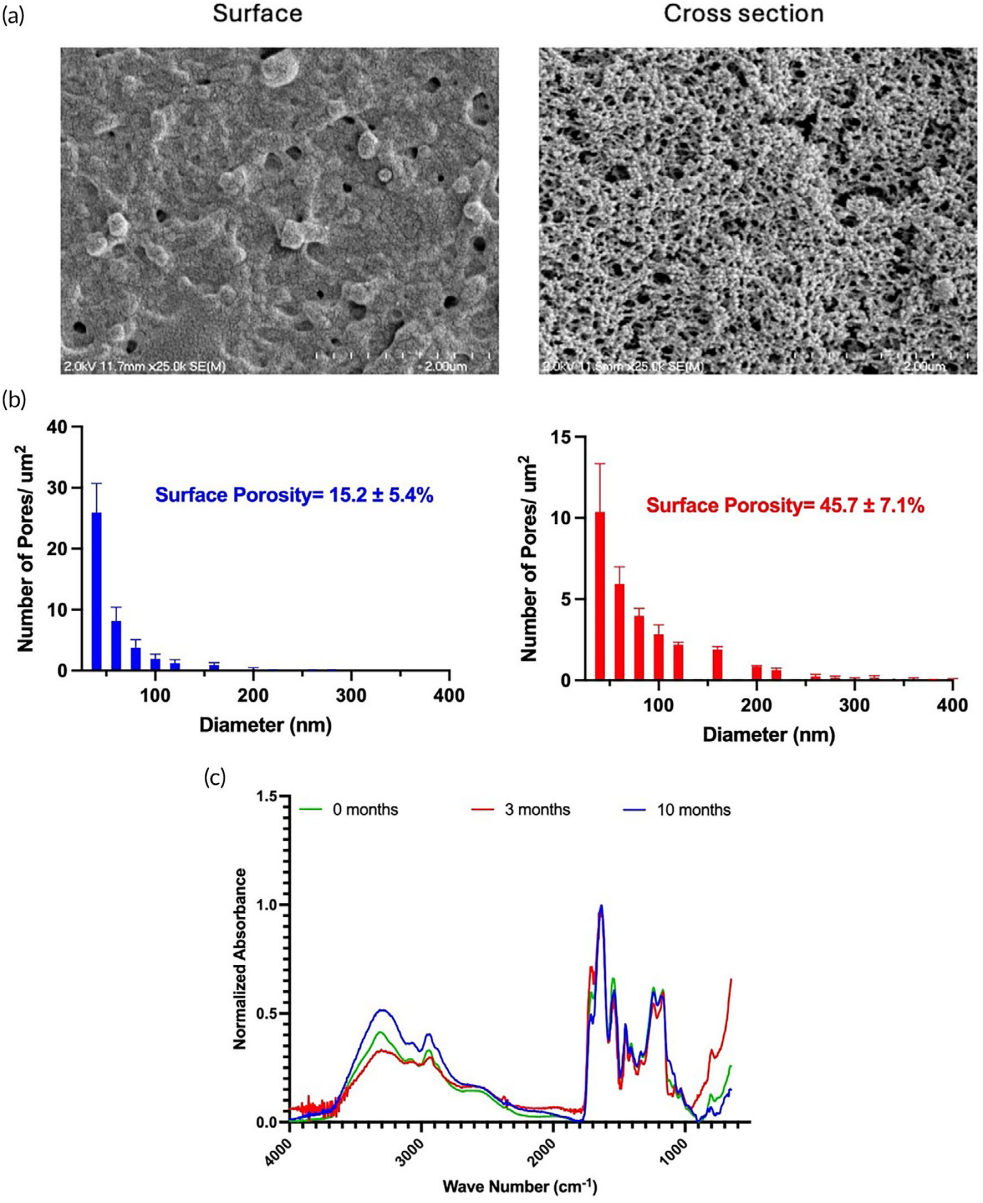
Characterization of skin meshes: (a) Representative images of scanning electron microscopy (SEM) of skin meshes to assess porosity of the meshes. Higher porosity of the mesh is observed in the cross section of the meshes than the surface. (b) Quantification of pore size distribution using ImageJ. (*n* = 3 per experimental group). (c) FTIR spectra comparing fresh 3D‐printed skin meshes with air‐dried meshes over time, demonstrating no chemical degradation during storage. Data is represented in mean ± SD.

The surface and cross‐sectional porosity of the auxetic skin mesh was examined using scanning electron microscopy (SEM). Prior to imaging, the meshes were dried using critical point drying to preserve structural integrity. As shown in Figure 3a, the SEM images reveal a uniform microstructure across the skin mesh. Pore sizes on the outer surface range from 40 to 760 nm, while those within the cross‐section range from 20 to 3300 nm. Due to the use of digital light processing (DLP) printing, the mesh exhibits a smoother outer surface, resulting in a relatively low surface porosity of approximately 15 ± 5.4% (Figure 3b). In contrast, the internal structure of the mesh displays a significantly higher surface porosity of 45.7 ± 7.1% (Figure 3b).

To evaluate potential chemical degradation, FTIR spectra of air‐dried skin meshes stored for 3 and 10 months were compared to freshly printed meshes (Figure 3c). The spectra showed no new peaks or peak shifts with storage time, indicating that the chemical composition of the meshes remained stable without detectable degradation products.

### 3.3 Loading and release kinetics of drug carriers

We have previously demonstrated that GelMA‐ACA‐based matrices are capable of loading EVs and releasing them in a sustained manner over time. In this study, we aimed to investigate the release kinetics of both EVs and LNPs, and to compare two different loading methods, incubation and rehydration, in terms of their loading capacities and release profiles.

**FIGURE 4:**
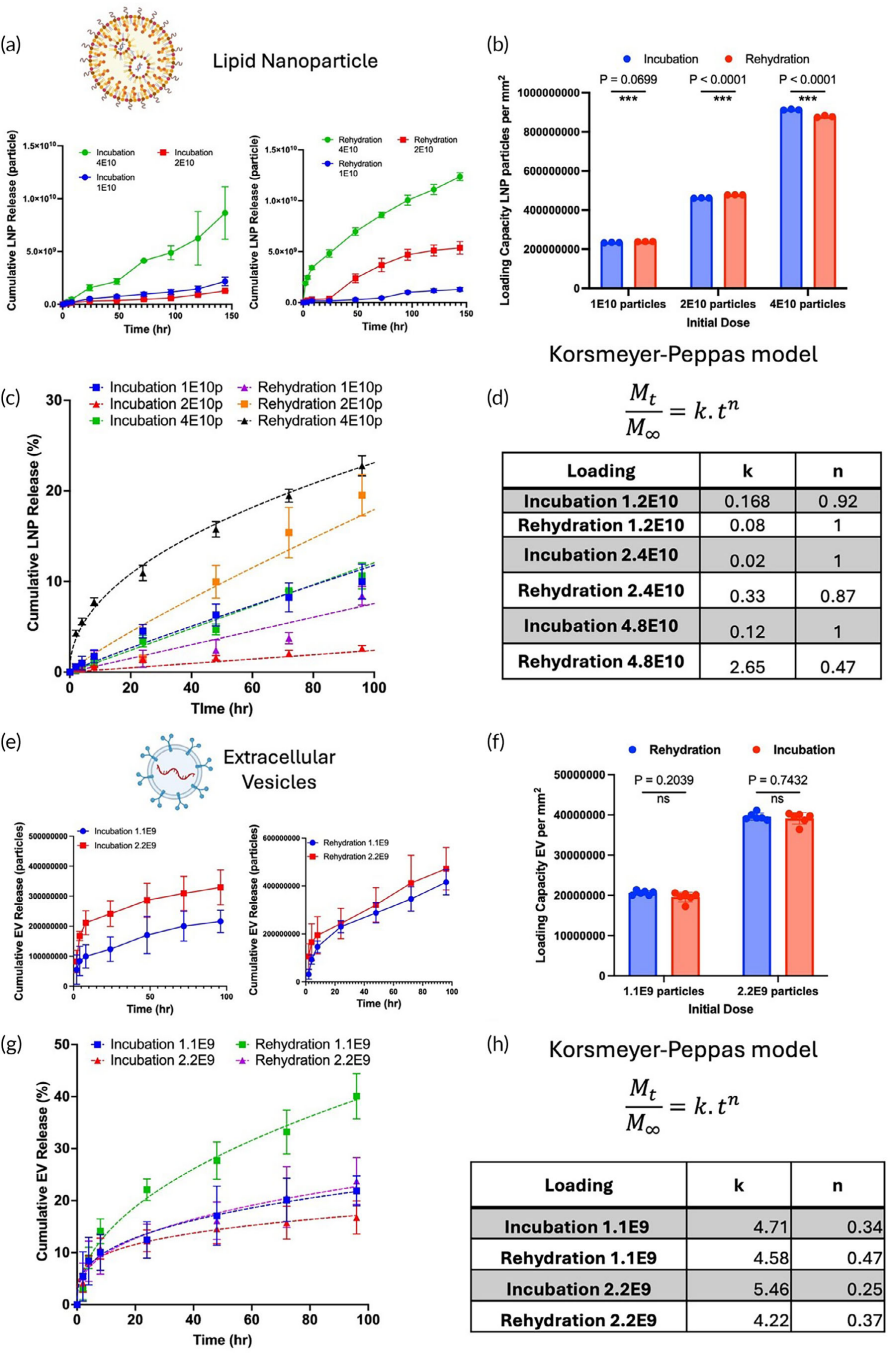
Characterization of loading and release of drug carriers: (a) Release of DiD‐labeled LNPs from skin meshes. (b) Comparison between loading capacity between incubation and rehydration method of loading skin meshes. One‐way ANOVA was performed, followed by Tukey's post hoc test for multiple comparisons (*n*= one mesh/condition; triplicate wells/timepoint). (c) % Cumulative release and release kinetics of LNPs from skin meshes. (d) Parameters from curves fitted to the Korsmeyer‐Peppas release profile. (e) Release of DiD labeled EVs from skin meshes. (f) Comparison of skin mesh loading efficiency: Incubation vs. rehydration method. One‐way ANOVA was performed, followed by Tukey's post hoc test for multiple comparisons (*n*= two meshes/condition; triplicate wells/timepoint). (g) % Cumulative release and release kinetics of EVs from skin meshes. (h) Parameters from curve fitted to Korsmeyer–Peppas release profile.

LNPs were synthesized using the Moderna formulation with the following lipid composition: SM‐102, DSPC, cholesterol, DMG‐PEG2000, and the lipophilic dye DiD at a molar ratio of 49:10:38.5:1.5:1. This yielded LNPs with the size of 156.1 ± 4.2 nm with a polydispersity index (PDI) of 0.10 ± 0.04 (Figure S1). First, we loaded DiD‐labeled LNPs onto our skin‐mimetic GelMA‐ACA meshes (Figure 4a). As shown in Figure 4b, the meshes successfully loaded LNPs. The incubation method demonstrated a superior loading capacity, though the difference was minimal. Release data were fitted to the Korsmeyer–Peppas model, and in almost all cases, the release exponent (n) was found to be near 1, indicating that the release mechanism was primarily governed by polymer swelling and erosion. (Figure 4c,d). The influence of diffusion appeared to be less pronounced, likely due to electrostatic interactions between the ionizable lipids and the carboxylic acid groups of polyacrylic acid. These interactions may have hindered free diffusion, thereby favoring a release mechanism dominated by polymer swelling and erosion.

Similarly, for EVs, we used DiD‐labeled vesicles to assess their release from the GelMA‐ACA matrix (Figure 4e). As shown in Figure 4f, no statistically significant difference in loading capacity was observed between the incubation and rehydration methods across all tested doses. Fitting the release data to the Korsmeyer–Peppas model yielded n values less than 0.5, consistent with diffusion as the dominant release mechanism (Figure 4g,h).

Interestingly, for the EV experiments, the *k* values were slightly lower and n values were consistently higher for the rehydration group compared to the incubation group. This trend may be attributed to the formation of denser polymer networks during the dehydration process, which likely increases the tortuosity of the diffusion path, as supported by our mechanical testing data.

### 3.4 Loading and release kinetics of small and large molecules

To evaluate the loading and release behavior of protein and small molecule therapeutics, we used FITC‐conjugated ovalbumin (OVA‐FITC) and Texas Red‐conjugated gentamicin (Gm‐TR), respectively. Both were loaded into the GelMA‐ACA meshes using either the incubation or rehydration method, as previously described.

**FIGURE 5:**
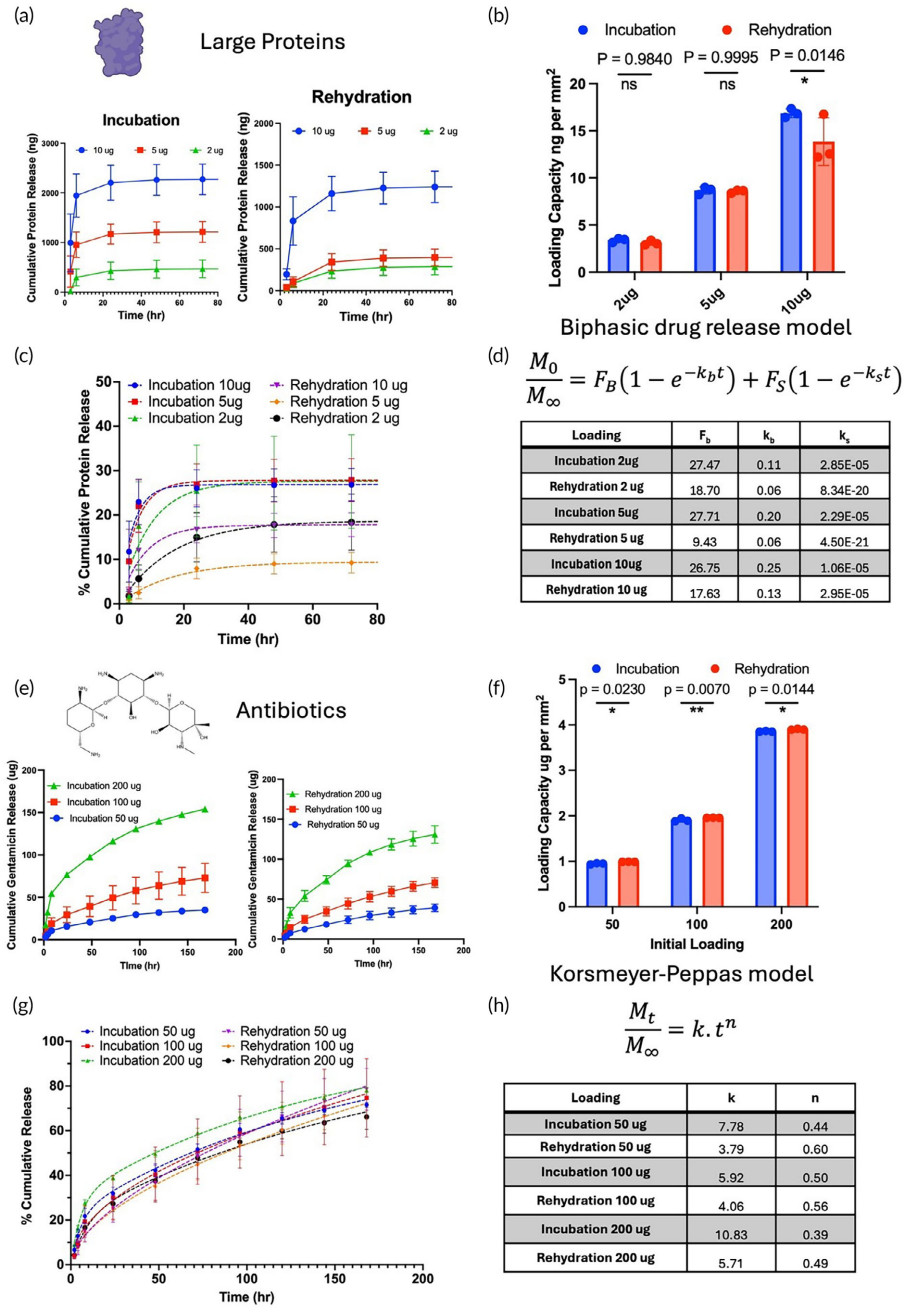
Characterization of loading and release of small and large molecules from skin meshes: (a) Release of OVA‐FITC from skin meshes. (b) Comparison between loading capacity between incubation and rehydration method of loading skin meshes. One‐way ANOVA was performed, followed by Tukey's post hoc test for multiple comparisons (*n*= three meshes/condition; triplicate wells/timepoint). (c) % Cumulative release and release kinetics of OVA‐FITC from skin meshes. (d) Parameters from curves fitted to a biphasic drug release model. (e) Release of gentamicin‐texas red from skin meshes. (f) Comparison of skin mesh loading efficiency: Incubation vs. rehydration method. One‐way ANOVA was performed with Tukey's post hoc test from multiple comparisons. (g) % Cumulative release and release kinetics of Gentamicin from skin meshes (*n*= three meshes/condition; triplicate wells/timepoint). (h) Parameter from curve fitted to Korsmeyer–Peppas release profile.

For the protein cargo (OVA‐FITC) (Figure 5a), loading capacity was comparable between the two methods at lower initial doses. However, at higher doses, the rehydration method resulted in significantly lower loading compared to incubation (Figure 5b). Release profiles for OVA‐FITC did not fit the Korsmeyer–Peppas model due to a pronounced initial burst release, resulting in a biphasic release pattern. Consequently, the data were fitted to a biphasic release model (Figure 5c,d), characterized by an initial burst phase followed by a sustained release phase. In this model, *Fb* and *Fs* represent the fractions of drug released during the burst and sustained phases, respectively, while *kb* and *ks* denote the corresponding release rate constants. The burst release is attributed to protein localized near or on the surface of the mesh, whereas the sustained release likely originates from protein embedded within the hydrogel matrix. Notably, at lower doses (2 and 5 μg), the sustained release rate (*ks*) was significantly lower for the rehydration method compared to incubation. This may be due to the formation of a denser polymer network during rehydration sequestering protein molecules within the matrix, as previously discussed.

Next, we loaded gentamicin onto the skin meshes and studied the release (Figure 5e). In the case of the small molecule gentamicin, no significant differences in loading capacity were observed between the two methods (Figure 5f). The release profiles followed a classical single‐phase release and were well described by the Korsmeyer–Peppas model. The release exponent *n* in some groups was >0.5, suggesting a combination of Fickian diffusion and polymer matrix swelling. This behavior is likely due to the small molecular size of gentamicin, which allows it to access smaller pores that become more available for release as the matrix swells. Consistent with previous observations, the *k* value decreased and the *n* value increased when transitioning from incubation to rehydration, further supporting the hypothesis that rehydration leads to a denser network that alters release dynamics (Figure 5g,h).

The observed differences in release behavior between protein and small molecule therapeutics may be attributed to size‐dependent diffusion into the matrix. Larger proteins are likely excluded from smaller pores and thus remain more surface‐associated, contributing to the initial burst release. In contrast, smaller molecules can penetrate deeper into the matrix, thereby reducing the initial burst release.

### 3.5 Release and functional activity of PDGF‐BB from skin meshes

**FIGURE 6:**
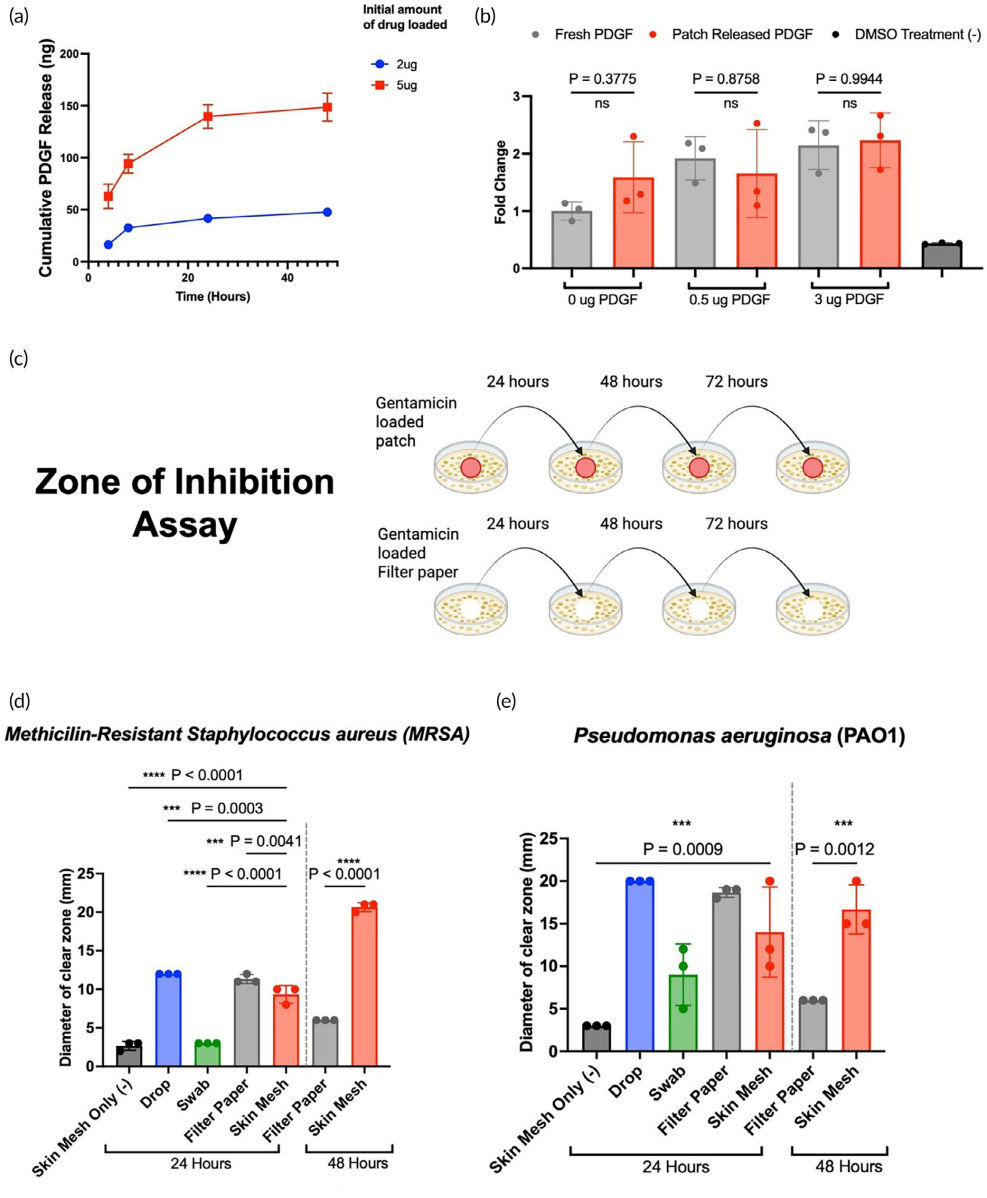
Release and stability of PDGF and gentamicin loaded onto skin meshes: (a) Release of PDGF‐BB from meshes over 48 h assessed via ELISA. (b) Stability of PDGF‐BB on skin meshes assess via NIH 3T3 proliferation assay. One‐way ANOVA was performed with Tukey's post hoc test from multiple comparisons (*n* = 3 per experimental group). (c, d) Zone of inhibition assay against (c) *Methicillin‐Resistant Staphylococcus aureus* (MRSA), and (d) *Pseudomonas aeruginosa* (PAO1) at 24 and 48 h. Gentamicin loaded skin mesh (red) were compared to skin mesh only (black), direct drop application (blue), swab application (green), and filter paper soaked in gentamicin (gray). One‐way ANOVA was performed followed by Sidak's post hoc test for multiple comparisons (*n* = 3 per experimental group). All data represented as mean ± SD.

Following our previous demonstration that proteins can be successfully loaded onto the skin mesh, we next evaluated whether the released protein remains stable and functionally active. To assess this, we loaded murine PDGF‐BB growth factor onto the mesh using a rehydration method. Two initial doses, 5 and 2 μg, were applied to the mesh, which was then incubated in PBS (release media) to allow for protein release. The release profile was quantified using ELISA. Over the first 48 h, we observed a sustained release of PDGF‐BB from the skin meshes, indicating effective delivery kinetics (Figure 6a).

To determine whether the released PDGF‐BB retained its biological activity, we assessed its ability to promote fibroblast proliferation which is a key mechanism by which PDGF‐BB accelerates wound healing through collagen production and tissue remodeling. NIH 3T3 fibroblast cells were treated with PDGF‐BB released from the mesh and compared to cells treated with freshly prepared PDGF‐BB.

Our results showed that the released PDGF‐BB remained functionally active, enhancing fibroblast proliferation. Specifically, proliferation increased by approximately 1.7‐ and 2.2‐fold in the 0.5 and 3 μg release groups, respectively, compared to untreated controls (Figure 6b). Importantly, there was no statistically significant difference in proliferative efficacy between the mesh‐released PDGF‐BB and the freshly prepared PDGF‐BB, confirming the stability and bioactivity of the released protein.

### 3.6 Release and functional activity of gentamicin from skin meshes

To evaluate the antibacterial efficacy of gentamicin released from the skin mesh, we performed zone of inhibition assays. Building on our previous findings that the mesh enables sustained release of gentamicin, we aimed to determine whether the released antibiotic remains effective against bacterial pathogens.

Given gentamicin's broad‐spectrum activity, we tested its efficacy against Gram‐positive methicillin‐resistant *Staphylococcus aureus* (MRSA) and Gram‐negative *Pseudomonas aeruginosa* (PAO1), two pathogens commonly found in diabetic wound infections.^23^, ^24^, ^25^ The gentamicin‐loaded mesh was compared to three other delivery methods: direct drop application, swab application, and filter paper soaked in gentamicin (Figure 6c).

On day 1, the gentamicin‐loaded mesh produced a significant zone of inhibition against MRSA, outperforming the swab method (Figure 6d). Although the drop and filter paper methods showed slightly larger zones initially, which is likely due to immediate drug release, the mesh demonstrated superior sustained activity. When transferred to a fresh agar plate on Day 2, the mesh continued to inhibit bacterial growth effectively, whereas the filter paper showed diminished activity. This suggests that the filter paper released most of its gentamicin on Day 1, while the mesh provided a more controlled, prolonged release.

A similar trend was observed with PAO1, further supporting the mesh's ability to maintain antibacterial efficacy over time through sustained drug release (Figure 6e). The mesh displayed 3.5‐fold enhanced performance in the MRSA group and 2.8‐fold enhanced performance in the *P. aeruginosa* group, compared to the filter paper on day 2 with MRSA and *P. aeruginosa* groups, respectively.

### 3.7 Promotion of angiogenesis mediated by PDGF‐BB loaded skin mesh in a diabetic wound healing murine model

**FIGURE 7:**
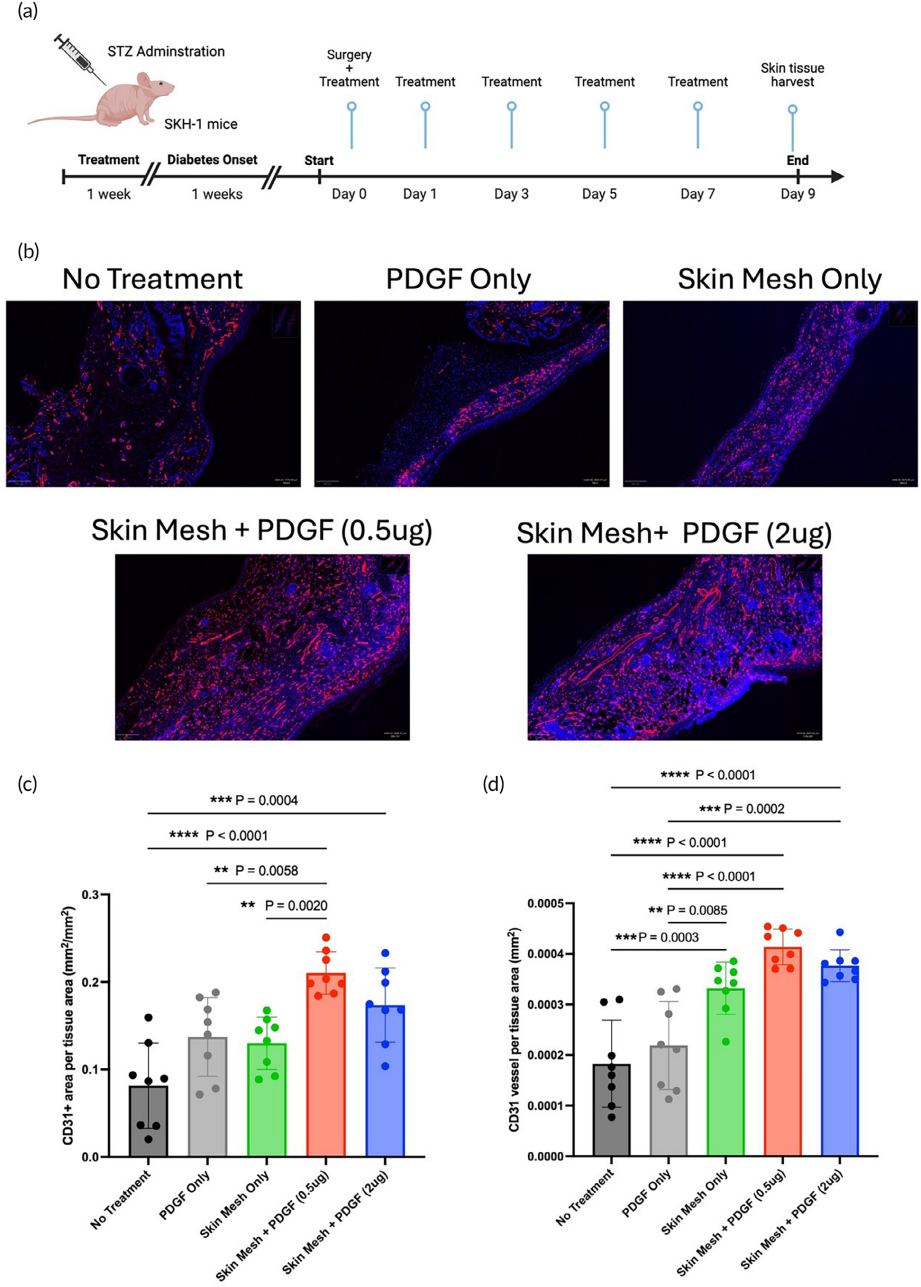
In vivo promotion of angiogenesis by PDGF‐BB loaded skin meshes in a diabetic mouse model: (a) Schematic depicting animal study model. (b) Immunofluorescence staining of CD31 endothelial cells and nucleus of the cells to study new blood vessel formation (*n* = 4 per group and 2 skin regions per animal). (c, d) Quantification of (c) CD31+ area and (d) number of CD31+ vessel in each group. Data represented as mean ± SD. One‐way ANOVA was performed with Tukey's post hoc test from multiple comparisons.

As shown in Figure 7a, diabetes was induced in mice using streptozotocin (STZ) administration, followed by 1 week of glucose monitoring. Mice with blood glucose levels exceeding 250 mg/dL were considered diabetic. To induce wounds, a 4 mm biopsy punch was used to create full‐thickness excisional wounds. The wounds were then treated with the respective interventions and covered with Tegaderm to prevent the mice from removing the treatments. The experimental groups included: No treatment (Tegaderm only), PDGF only (2 μg), Skin mesh only, and Skin mesh + PDGF (0.5 or 2 μg).

Treatments were reapplied every alternate day. At the conclusion of the study (Day 9), skin samples were collected for histological analysis (Figure 7b). Immunofluorescence staining using CD31 and DAPI demonstrated that the skin mesh combined with PDGF resulted in a significantly higher number of CD31‐positive blood vessels and a greater total vascular area compared to both the no‐treatment and PDGF‐only groups (Figure 7c,d). PDGF‐BB is known to promote angiogenesis, and the enhanced vascularization observed in the skin mesh + PDGF group suggests that sustained release of PDGF from the mesh may have contributed to improved angiogenic outcomes. These findings indicate that the delivery platform plays a critical role in modulating the therapeutic efficacy of PDGF‐BB in promoting neovascularization within the wound bed.

## 4 DISCUSSION

We have previously demonstrated the effectiveness of using auxetic skin meshes for various regenerative applications. These skin meshes, composed of gelatin‐based materials, are biocompatible, bioadhesive, ultraelastic, and capable of drug loading.^9^ In this study, we developed a shelf‐stable skin mesh that can be loaded via a simple dip‐and‐deliver method and demonstrated its versatile drug loading capacity across a wide spectrum of therapeutic classes, including small molecules, proteins, EVs, and LNPs. We further characterized the release profiles of these agents and evaluated their biological efficacy.

To enhance the clinical translatability of our skin mesh technology, we explored two drying methods to create shelf‐stable formulations: air drying and freeze‐drying. Mechanical characterization via stress–strain analysis revealed that both drying methods increased the stiffness of the skin meshes, as indicated by a higher Young's modulus compared to the unprocessed skin mesh. This observation is consistent with a previous study that reported increased material stiffness, fracture stress, and fracture upon dehydration due to the formation of additional hydrogen bonds between the polymer chains.^26^ This is largely attributed to an increase in contact and reduced mobility of the polymer chain when the meshes are dried, thus increasing hydrogen bonding.^26^ This process increases the fatigue threshold, which can be beneficial for skin applications as the skin is under constant repetitive motion that can mount fatigue onto the mesh over time.

Importantly, bioadhesion testing showed that air drying did not significantly alter the adhesive properties of the meshes, whereas freeze‐drying led to a marked reduction in adhesion. This reduction may be attributed to microfractures formed during the freezing process, likely due to ice crystal formation, as previously reported.^27^ Based on these findings, air‐dried meshes were selected as the optimal format for drug loading and storage.

We further demonstrated that the skin mesh is compatible with a wide range of therapeutic agents, including lipid nanoparticles, extracellular vesicles, proteins, and small molecules. Drug release studies revealed distinct release kinetics depending on the class of therapeutic. Given the porosity of the meshes and high density of carboxylic acid groups, the loading of these drugs occurs via free drug diffusivity through the hydrogel and electrostatic interactions between drug and matrix. Gentamicin is a small drug with a size smaller than the pore sizes within the hydrogel, indicating that the drug can freely diffuse through the hydrogel. Furthermore, gentamicin is an aminoglycoside antibiotic that carries a positive charge at physiological pH (≈7), enabling electrostatic interactions with the negatively charged functional groups within the hydrogel matrix.^28^ This allows the gentamicin drug to be loaded. Next OVA is ~3 nm in diameter allowing it to freely diffuse through the hydrogel matrix, however due to its net negative charge at physiological pH repulsive forces likely led to burst release.^29^

LNPs and EVs measured approximately 150 nm in diameter, exceeding the size of 96% of surface pores and 87% of internal pores within the matrix. This indicates that these particles are not freely diffusible within the hydrogel matrix and thus mainly rely on hydrogen bonding and electrostatic interaction with the matrix.^9^ The LNPs were formulated using cationic lipids, which may have further aided matrix‐drug electrostatic interactions, allowing LNP to be loaded onto the matrix.

EV release was primarily governed by diffusion, as inferred from the Korsmeyer Peppas model fitting. Whereas gentamicin, when loaded via rehydration, exhibited a more complex release mechanism involving both diffusion and polymer swelling. This difference may be due to the smaller molecular size of gentamicin, which allows it to penetrate deeper into the matrix during rehydration, becoming entrapped in smaller pores that require swelling for release. In contrast, larger drug carriers like EVs are excluded from these pores, resulting in diffusion‐dominated release.

On the other hand, protein release exhibited a biphasic profile, characterized by a pronounced initial burst followed by a sustained release phase. The sustained release of OVA; however, was only observed up to 48 h, after which no further release was observed. This behavior may be attributable to low matrix–protein interactions, which facilitate rapid diffusion and release during the initial phase. The low‐protein interaction could be explained due to the net negative charge of OVA at pH 7, creating repulsion between carboxylate groups and OVA.^30^

LNPs exhibited a distinctive release profile primarily governed by polymer relaxation. Unlike EVs, LNPs contain ionizable lipids such as SM‐102, which features a tertiary amine group. This cationic amine can form electrostatic interactions with the anionic carboxylic acid groups in poly(acrylic acid), thereby restricting diffusion. Eriksson et al. demonstrated a similar phenomenon, where the cationic antimicrobial octenidine dihydrochloride interacted electrostatically with anionic poly(d,l‐lactide‐co‐glycolide), resulting in a reduction in effective diffusivity and enabling prolonged, sustained release.^31^

To evaluate the therapeutic relevance of our platform, we selected two clinically relevant drugs for diabetic wound healing: PDGF‐BB and gentamicin. PDGF‐BB is a well‐established growth factor that promotes angiogenesis and fibroblast proliferation, both of which are impaired in diabetic wounds.^32^ Its clinical relevance is underscored by the FDA‐approved topical PDGF formulation, Regranex®.^33^ Gentamicin, a broad‐spectrum antibiotic, was chosen to address the high risk of infection in diabetic wounds, which is exacerbated by hyperglycemia and impaired immune responses.^34^ Our in vitro studies confirmed that PDGF‐loaded meshes enhanced fibroblast proliferation, while gentamicin‐loaded meshes provided sustained release over 7 days and effectively inhibited the growth of MRSA and *P. aeruginosa*, two pathogens commonly associated with diabetic wound infections.^23^, ^24^, ^25^

Hyperglycemia impairs angiogenesis in diabetic foot ulcers (DFUs) through multiple mechanisms. Under hypoxic conditions, elevated glucose levels disrupt the activity of hypoxia‐inducible factor‐1α (HIF‐1α), leading to reduced expression of key pro‐angiogenic mediators such as vascular endothelial growth factor (VEGF) and stromal‐derived factor‐1α (SDF‐1α).^35^ Additionally, hyperglycemia enhances reactive oxygen species (ROS) production, which further inhibits neovascularization.^36^ It also directly suppresses the proliferation and function of endothelial progenitor cells (EPCs), critical for tubule formation.^37^, ^38^ Enhancing angiogenesis in DFUs can restore oxygen and nutrient delivery, thereby promoting cell migration, matrix deposition, epithelialization, and ultimately wound closure.^39^ PDGF‐BB is an FDA‐approved drug shown to promote angiogenesis via stabilization of vessel through pericyte recruitment as well as stimulate extracellular matrix production crucial in supporting vessel wall structure and integrity.^40^, ^41^, ^42^ Thus, we assessed the in vivo effects on angiogenesis using an STZ‐induced diabetic mouse model. Meshes loaded with PDGF‐BB significantly enhanced angiogenesis within the wound bed, as evidenced by increased CD31+ vessel density. These findings suggest that the mesh effectively delivers PDGF‐BB and promotes angiogenesis.

Overall, our auxetic skin mesh platform demonstrates high tunability, biocompatibility, and versatility in drug loading and release. The ability to rehydrate the meshes at the point of care, while maintaining sustained release profiles for diverse therapeutic agents, makes this technology highly translatable. The platform's compatibility with proteins, small molecules, and nanoparticles opens the door to synergistic drug combinations, such as PDGF‐BB and gentamicin, offering a promising strategy for enhancing diabetic wound healing while preventing infection.

## 5 CONCLUSION

The present study expands the range of therapeutics that can be delivered using our auxetic skin mesh platform. To enhance the clinical translatability of this technology, we investigated the feasibility of drying the mesh for shelf‐stable storage. We systematically evaluated the impact of dehydration followed by rehydration on the mesh's mechanical integrity, chemical properties, and drug release kinetics. Furthermore, we demonstrated the therapeutic potential of our system in a diabetic wound healing model, where impaired tissue regeneration is a major clinical challenge. Delivery of PDGF‐BB via the auxetic mesh significantly enhanced angiogenesis within the wound bed, highlighting the mesh's capability as a versatile wound care platform.

## AUTHOR CONTRIBUTIONS

The study concept and experimental design were developed by Ameya P. Chaudhari and Juliane Nguyen. Experiments were conducted and data collected by Ameya P. Chaudhari, Parmiss Khosravi, Zajeba Tabashsum, Hattie E. Hensley, Claire J. Wang, Emilie A. Moses, Samantha Harris, Anthony Hazelton, Palas B. Tiwade, and Rachel VanKeulen‐Miller. Data analysis was carried out by Ameya P. Chaudhari, Parmiss Khosravi, and Zajeba Tabashsum. The original manuscript was drafted by Ameya P. Chaudhari, with critical review provided by Owen S. Fenton, Sergei S. Sheiko, Sarah E. Rowe, and Juliane Nguyen. Ameya P. Chaudhari and Juliane Nguyen edited and made final revisions to the manuscript.

## CONFLICT OF INTEREST STATEMENT

Juliane Nguyen and Ameya Chaudhari are inventors on a pending patent application related to the technology described in this manuscript.

## ETHICS STATEMENT

No ethical considerations were required for this study.

## Supporting information


**DATA S1.** Supporting Information.

## Data Availability

The data that support the findings of this study are available from the corresponding author upon reasonable request.
