# Supplementary material for: Shelf‐stable, ready‐to‐use therapeutic patches: Dip‐and‐deliver solutions for personalized wound care
Source: Bioeng Transl Med. 2026 Mar 23;11(4):e70131. doi: 10.1002/btm2.70131 (PMC13327606; doi:10.1002/btm2.70131)
Supplement: Supplementary file 1 — DATA S1. Supporting Information. [file BTM2-11-e70131-s001.docx]

**Supplementary Information**

**Shelf-stable, Ready-to-Use Therapeutic Patches: Dip-and-Deliver Solutions for Personalized Wound Care**

Ameya P. Chaudhari^1^, Parmiss Khosravi^2^, Zajeba Tabashsum^3^, Hattie E. Hensley^2^, Claire J. Wang^4^, Emilie A. Moses^4^, Samantha Harris^1^, Anthony Hazelton^5^, Palas B. Tiwade^1^, Rachel VanKeulen-Miller^5^, Owen S. Fenton^1^, Sergei S. Sheiko^4^, Sarah E. Rowe^3^, Juliane Nguyen^1,2,6*^

1. Division of Pharmacoengineering and Molecular Pharmaceutics, Eshelman School of Pharmacy, University of North Carolina at Chapel Hill, Chapel Hill, NC, 27599, USA
2. Lampe Joint Department of Biomedical Engineering, University of North Carolina at Chapel Hill, Chapel Hill, NC, 27599, USA
3. Department of Microbiology and Immunology, University of North Carolina-Chapel Hill, Chapel Hill
4. Department of Chemistry, University of North Carolina at Chapel Hill, Chapel Hill, NC, 27599, USA
5. Department of Pharmacology, School of Medicine, University of North Carolina at Chapel Hill, Chapel Hill, NC, 27599, USA
6. UNC Mcllister Heart Institute, University of North Carolina at Chapel Hill, Chapel Hill, NC, 27599, USA

***Corresponding Author**

**Email: julianen@email.unc.edu**

**Juliane Nguyen, PhD, FCRS, FAAPS, FAIMBE**Professor and Vice Chair
Director of Graduate Admissions – DPMP
Division of Pharmacoengineering and Molecular Pharmaceutics
Eshelman School of Pharmacy
Adjunct Professor in  Biomedical Engineering (BME)
Member of the UNC Lineberger Cancer Center
Member of the UNC McAllister Heart Institute
University of North Carolina at Chapel Hill

**
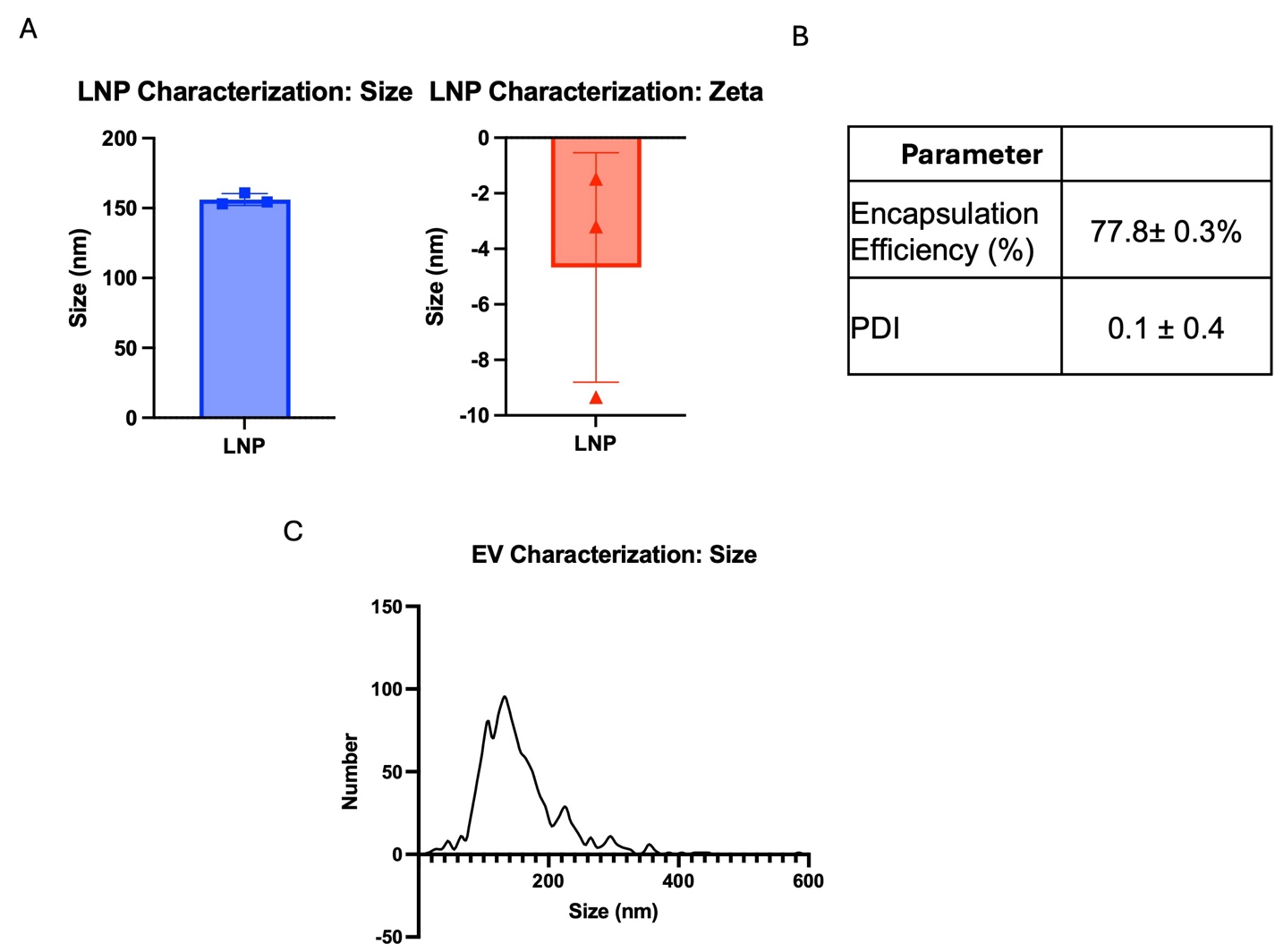
**

**Supplementary Figure 1: Characterization of drug carriers** A) Assessment of size and zeta potential of LNPs. B) Reporting Encapsulation Efficiency (%) and polydispersity index of LNP. C) Assessment of size distribution of EVs.
